# Neutrophil extracellular traps in relationship to efficacy of systemic therapy for metastatic renal cell carcinoma

**DOI:** 10.1002/cam4.6748

**Published:** 2023-11-28

**Authors:** Ruiyang Xie, Bingqing Shang, Hongzhe Shi, Xingang Bi, Yan Song, Wang Qu, Hongsong Bai, Linjun Hu, Jie Wu, Honglei Cui, Gan Du, Lei Guo, Shan Zheng, Jianming Ying, Changling Li, Jianhui Ma, Aiping Zhou, Jianzhong Shou

**Affiliations:** ^1^ Department of Urology Peking University Third Hospital Beijing China; ^2^ Department of Urology, National Cancer Center/National Clinical Research Center for Cancer/Cancer Hospital Chinese Academy of Medical Sciences and Peking Union Medical College Beijing China; ^3^ Department of Medical Oncology, National Cancer Center/National Clinical Research Center for Cancer/Cancer Hospital Chinese Academy of Medical Sciences and Peking Union Medical College Beijing China; ^4^ Department of Urology, Cancer Hospital of Huanxing Beijing China; ^5^ Department of Pathology, National Cancer Center/National Clinical Research Center for Cancer/Cancer Hospital Chinese Academy of Medical Sciences and Peking Union Medical College Beijing China

**Keywords:** efficacy prediction, metastatic clear cell renal cell carcinoma, neutrophil extracellular traps, systemic therapy

## Abstract

**Background:**

The efficacy of systemic therapy regimens, such as immune checkpoint inhibitors and tyrosine kinase inhibitors (IO‐TKI) and targeted therapy, for metastatic clear cell renal cell carcinoma (ccRCC) remains unpredictable due to the lack of effective biomarkers. Neutrophil extracellular trap (NET) plays an important role in promoting ccRCC. This study explores the NET predictive value of the efficacy in metastatic ccRCC.

**Methods:**

In this retrospective study, patients with metastatic ccRCC who received targeted drugs and IO‐TKI were included. Immunofluorescence staining was utilized to quantify the levels of tissue NETs through cell counts of H3Cit(+) and MPO(+) cells.

**Results:**

A total of 183 patients with metastatic ccRCC were enrolled, including 150 patients who received TKIs and 33 patients who received IO‐TKI. The levels of NETs in tumor tissue were significantly higher than in para‐tumor tissue (*p* < 0.001). In terms of predicting drug efficacy, a correlation between NET levels and progression‐free survival (PFS) was observed in the TKI with metachronous metastasis group (HR 1.73 [95% CI 1.02–2.91], log‐rank *p* = 0.037), while no correlation was observed in the TKI with synchronous metastasis group and IO‐TKI group. Regarding overall survival (OS), activated NET levels were associated with poor OS in both TKI (HR 1.60 [95% CI 1.05–2.43], log‐rank *p*  = 0.017) and IO‐TKI group (HR 4.35 [95% CI 1.06–17.82], log‐rank *p* =0.047). IMDC score (HR 1.462 [95% CI 1.030–2.075], *p* = 0.033) and tumor tissue NET levels (HR 1.733 [95% CI 1.165–2.579], *p* = 0.007) were independent prognostic risk factors for OS in patients with metastatic ccRCC.NET level was associated with poor OS in both TKI (HR 1.60 [95% CI 1.05–2.43], log‐rank *p*  = 0.017).

**Conclusions:**

The active NET levels in tumor tissue can predict drug efficacy in patients with metastatic ccRCC who received systemic therapy. Elevated levels of NETs in tumor tissue were also associated with poor efficacy in OS.

## Graphical abstract

Neutrophil extracellular trap (NET) was activated in primary ccRCC tumor tissue. The lower level of NET was associated with better PFS in patients treated with TKI and better OS in both TKI and IO‐TKI group. NET could be the mechanism of neutrophil promoting tumor, and NET formation in ccRCC tumor tissue was associated with poor clinical outcomes.

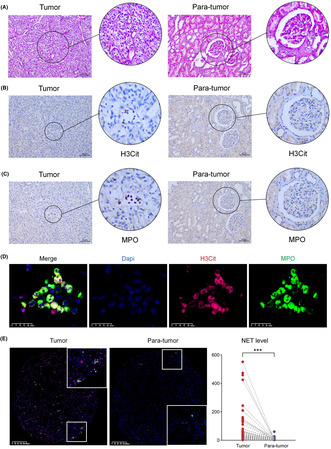

## 1 INTRODUCTION

Neutrophils, as the most enriched subtype of white blood cells in humans, serve an important role in the adaptive immune and tumorigenesis.^1^ Neutrophil lysis releases network structures composed of nuclear chromatin and antimicrobial granules, known as neutrophil extracellular trap (NET).^2^ NETs contain DNA‐coated molecules such as neutrophil elastase (NE), myeloperoxidase (MPO), histones, etc.^3^, ^4^ Some specific proteins are used as quantifying biomarkers in human tissue and peripheral blood: Citrullinated Histone H3 (H3Cit), MPO, MPO‐DNA complex, and NE‐DNA complex.^5^, ^6^ The tumor‐promoting or anti‐tumor role of NET can be altered, depending on the interaction of the immune system and tumor microenvironment. Previous research suggested that NET promoted tumorigenesis in the tumor microenvironment and was associated with metastasis and decreased treatment response in multiple malignancies (bladder cancer, Lewis lung carcinoma, and melanoma).^7^, ^8^, ^9^ By immunofluorescence staining, Park et al. discovered NET presence in both primary lesions and metastasis of breast cancer.^10^ For patients with colorectal cancer liver metastasis, the enrichment of tumor‐associated neutrophils (TAN) and NET was higher than that of normal liver tissue as well.^11^ Furthermore, serum baseline level of MPO‐DNA was significantly higher in tumor patients than in healthy population controls and was correlated with poor disease‐free survival (DFS) and overall survival (OS).^11^

Since neither tumor mutation burden (TMB) nor PD‐L1 correlated with immune checkpoint inhibitor (ICI) response,^12^ the effective biomarker in tissue for efficacy prediction is needed. Neutrophils have a great impact on the progression of kidney cancer because they reflect the intrinsic immune status. For the efficacy of immune checkpoint blockade, increased neutrophil‐lymphocyte ratio (NLR) was associated with worsened progression‐free survival (PFS).^13^ NLR is also an independent risk factor for poor prognosis in kidney cancer.^14^, ^15^ The International Metastatic Renal Cell Carcinoma Database Consortium (IMDC) proposed a scoring criteria to stratify the prognostic risk of metastatic renal cell carcinoma (RCC), which annotated elevated neutrophil count as a risk factor.^16^ IMDC risk group is used to screen population with favorable efficacy in clinical trials of immune checkpoint inhibitors with tyrosine kinase inhibitor (IO‐TKI) and targeted agents.^17^, ^18^ Neutrophils exert tumor‐promoting effects in a variety of mechanisms, including NET. The fibrin network and DNA framework released by NET arrest circulating tumor cells (CTCs) in kidney cancer, then help them escape from attack by the immune system and survive.^19^ In previous studies, we constructed gene sets based on NET and found that activated NET in pan‐cancer (lung adenocarcinoma, triple‐negative breast cancer, colon adenocarcinoma, and kidney renal clear cell carcinoma) indicated poor clinical outcomes.^20^ To date, the NET predictive value for the efficacy of systemic therapy in metastatic ccRCC remains unclear. In the present study, we collected tumor tissue specimens from patients with metastatic ccRCC who received targeted agents and IO‐TKI, then quantified NET in tumor tissue. As NET plays an important role in promoting ccRCC, this study explores the NET predictive value of the efficacy in metastatic ccRCC.

## 2 MATERIALS AND METHODS

### 2.1 Patients selection and study design

The study retrospectively enrolled patients with metastatic clear cell renal cell carcinoma (ccRCC) who received systemic therapy from January 1, 2006 to June 1, 2022. Systemic therapy regimens referred to vascular endothelial growth factor‐tyrosine kinase inhibitor (VEGF‐TKI) and PD‐1 immune checkpoint blockade with axitinib (IO‐TKI). We collected the clinicopathological data and specimen wax of the enrolled patients. The pathologic TNM stage of all patients was confirmed by pathologists according to the 2017 American Joint Committee on Cancer (AJCC) 8th Edition.^21^ This retrospective study was approved by the local Ethics Committee of the National Cancer Center and waived informed consent (Ethics Approval Number: NCC2020C‐122). Among the patients with metastatic ccRCC who received systemic therapy, the VEGF‐TKIs included sunitinib, pazopanib, and axitinib. PD‐1 inhibitors included pembrolizumab and tislelizumab. For the 33 patients who received second‐line IO‐TKI or TKI treatment, the prior treatments were regarding to sunitinib or pazopanib. The inclusion criteria for the cohort were as follows:

1. Histologically confirmed as ccRCC by pathologists
2. Previous first‐line/second‐line system therapy (VEGF‐TKI and IO‐TKI)
3. Available specimen wax archived in the Department of Pathology
4. Complete medical records, laboratory tests, imaging examinations, and pathological data

Patient exclusion criteria were as follows:

1. Patients enrolled in blinded clinical trials
2. Patients with pathological initial diagnosis of ccRCC, but the report is suspicious
3. Other malignant tumors during drug treatment
4. History of hematology‐related diseases
5. Low‐quality slice staining
6. Co‐infection or short‐term use of antibiotics
7. Non‐recommended VEGF‐TKI in NCCN guidelines

**FIGURE 1:**
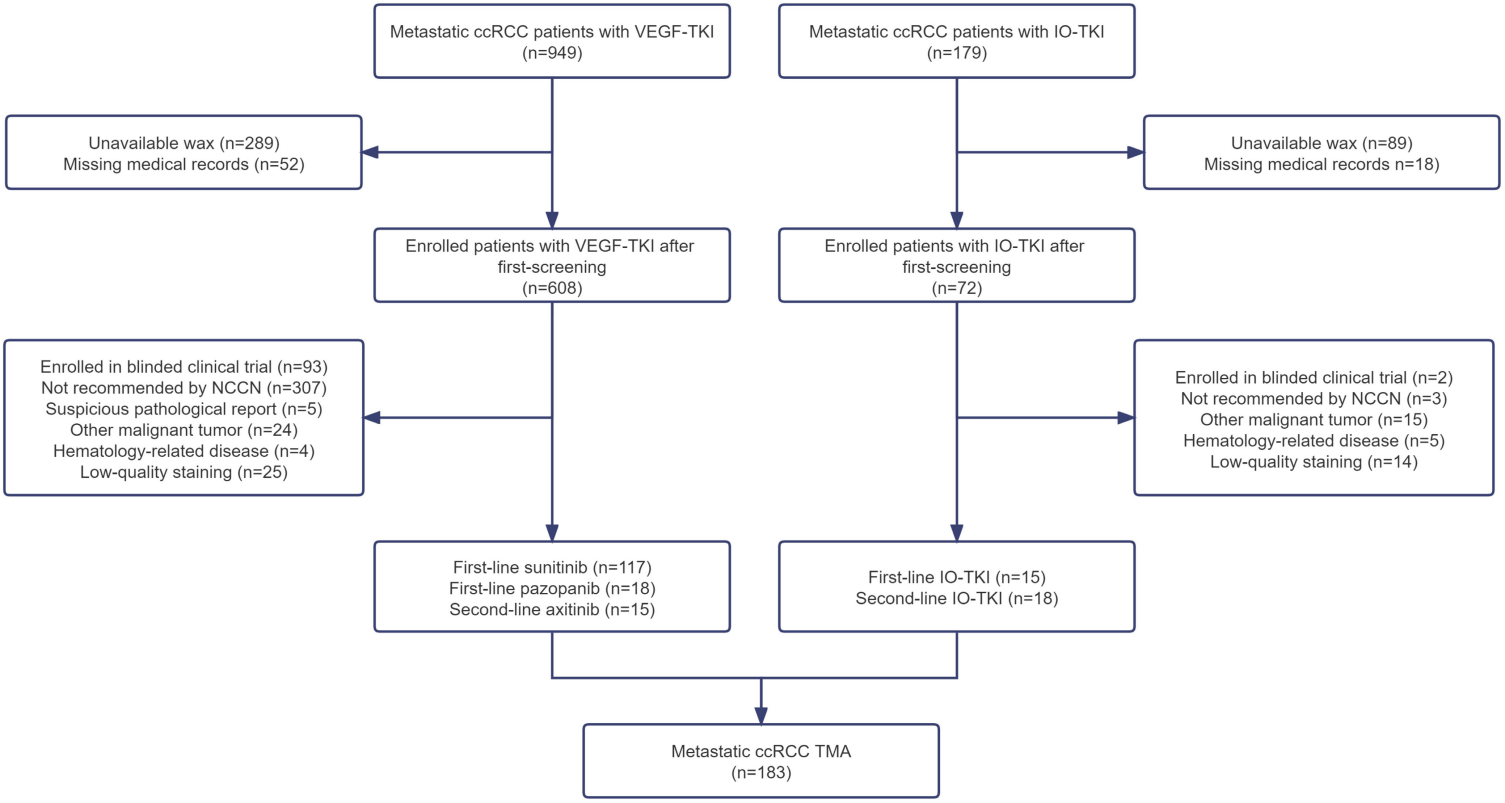
Workflow of patient screening for metastatic ccRCC.

As shown in Figure 1, a total of 949 patients received TKIs and 179 patients received IO‐TKI. Through the above screening criteria, 117 cases received first‐line sunitinib, 18 cases with first‐line pazopanib, 15 cases with second‐line axitinib, 15 cases with first‐line IO‐TKI, and 18 cases with second‐line IO‐TKI. A total of 183 metastatic ccRCC patients treated with systemic therapy were enrolled and analyzed as tissue microarray (TMA). For patients enrolled in the study, we assessed clinicopathologic data including sex, age at initial diagnosis, tumor diameter, TNM stage, histologic grade, presence or absence of renal venous tumor thrombus, PFS, OS, time from diagnosis to treatment, Karnofsky performance status (KPS), pretreatment NLR, and IMDC risk group. The cutoff value for NLR at 3.0 is validated in series of studies.^22^, ^23^, ^24^ PFS and OS are defined as the date on which the patients start systemic therapy until disease progression or death/last follow‐up respectively. For ccRCC patients treated with IO‐TKI, the objective response rate (ORR) was also evaluated by RECIST v1.1.^25^

### 2.2 TMA design and immunofluorescence staining

A blank wax block of 30*25*10 mm was selected for designing a microarray of 12*8 sites. For each patient, three tumor tissue sites and one para‐tumor tissue site were collected. A single TMA wax block consists of tissue from 18 to 22 patients. The constructed tissue array wax blocks were placed in a 55°C incubator for 10 min, then cooled at room temperature, and stored in a 4°C freezer. The tissue wax block was precooled at −10°C and fixed on a microtome for 4 μm continuous slices. Hematoxylin–eosin (HE) staining was performed to confirm specimens. TMAs were heated at 60°C for 1 h, dewaxed at room temperature, and washed for 30 min. 2.5% donkey serum (abs935, Absin Biotechnology) was used for incubation. For primary antibody incubation, 1:100 dilution of anti‐myeloperoxidase antibody (MAB3174, R&D Systems) and 1:100 dilution of anti‐histone H3 (citrulline R2 + R8 + R17) antibody (ab5103, Abcam) were used and incubated with TMA overnight at 4°C. For second antibody incubation, 1:200 dilution of Alexa Fluor 488 (711‐545‐152, Jackson ImmunoResearch) and 1:200 dilution of Alexa Fluor 594 (715‐585‐150, Jackson ImmunoResearch) were used. DAPI was used for nuclear staining (Boster Biological Technology), and a laser scanning microscope (KF‐PRO‐020‐HI, China) was used to observe and scan the TMA. Random 5 fields of view were captured for each site in K‐Viewer software. The co‐expression cell count of H3Cit(+) and MPO(+) was used to quantify tissue NET level. Two independent researchers measured, calculated, and averaged cell counts. Any discrepancies were discussed and solved by a senior researcher (J. Shou).

### 2.3 Statistics

The optimal cutoff values of NET level were determined using X‐tile 3.6.1.^26^ The statistical analyses were performed in SPSS 23.0 and GraphPad Prism 8.0.2, and the immunofluorescence was quantified in K‐Viewer 1.7.0. Counts and percentages were used for the features of the patient's clinicopathological data. The Mann–Whitney U test was used to compare the expression of NET in tumor tissue and para‐tumor tissue. PFS and OS survival were analyzed with the Kaplan–Meier method and log‐rank test, and the difference between groups was shown as hazard ratio (HR) and 95% confidence interval (CI). Statistical analysis was identified as significant when *p* < 0.05.

## 3 RESULTS

### 3.1 The association of NET level and clinicopathological data in metastatic ccRCC

**FIGURE 2:**
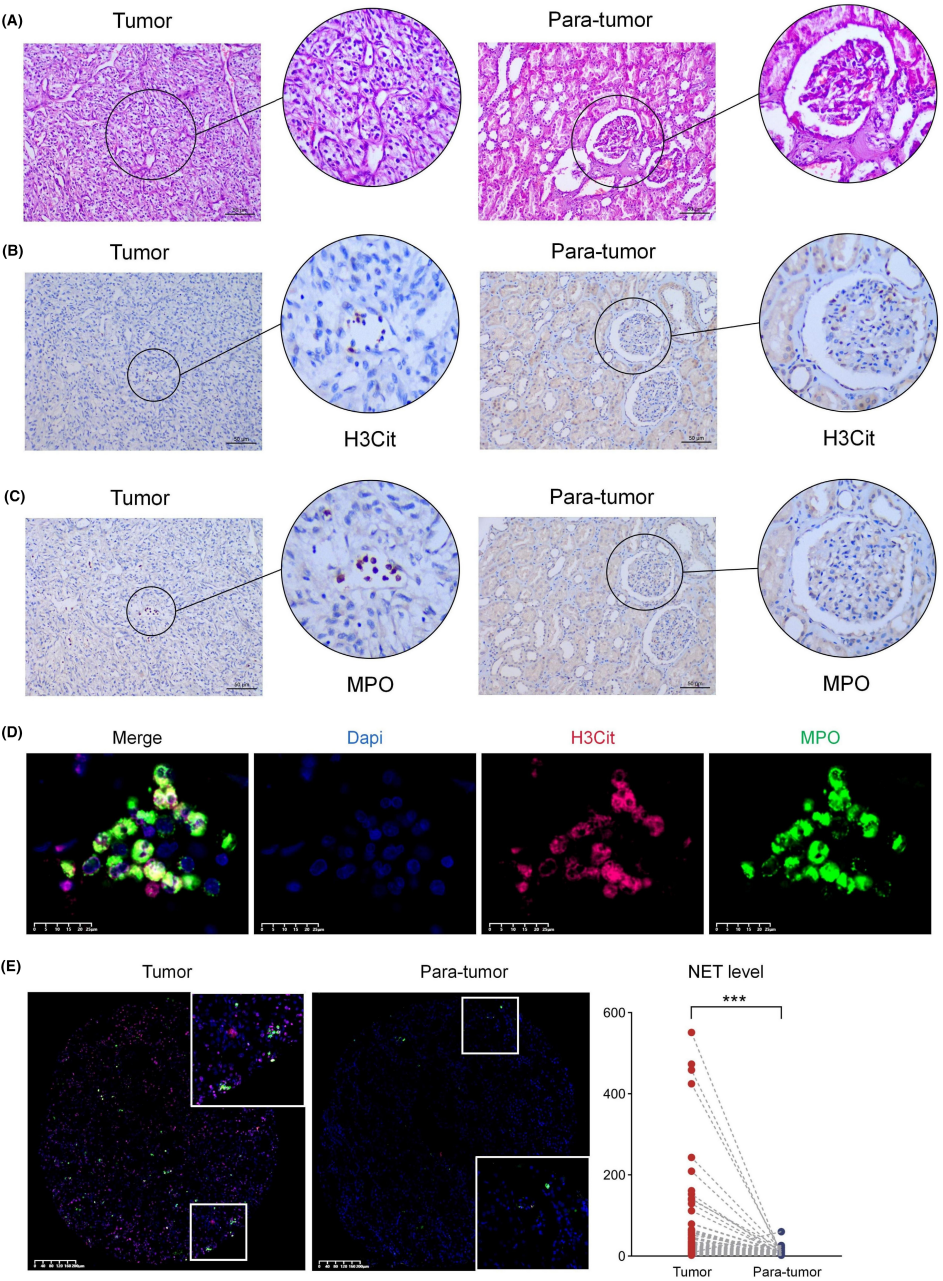
Pathological features and NET marker expression in metastatic ccRCC. (A) Tissue HE staining (B) Tissue H3Cit immunohistochemistry (IHC) staining (C) Tissue MPO IHC staining (80×, magnification 400×, scale bar: 200 μm) D. 800× magnified Immunofluorescence staining of NET in tumor tissue (scale bar: 25 μm) E. NET in matched tumor tissue and adjacent normal tissue (magnification 200× and 400×, scale bar: 50 μm, ***p < 0.001).

A total of 183 patients with metastatic ccRCC were enrolled in this study. As shown in Table 1, 135 patients (73.8%) received first‐line TKIs, 15 patients (8.2%) received second‐line TKIs, 15 patients (8.2%) received first‐line IO‐TKI, and 18 patients (9.8%) received second‐line IO‐TKI. Among all patients, 90 (49.1%) had synchronous metastases and 93 (50.9%) had metachronous metastases. For IMDC score, 40 patients (21.9%) were with favorable risk, 88 (48.1%) were with intermediate risk, and 55 (30.0%) were with poor risk. Nine slices of TMA were reviewed by pathologists with HE staining (Figure 2A), which included 508 tumor tissue sites and 138 para‐tumor tissue sites (Figure S1 **)**. To confirm the antibody efficacy, we first performed immunohistochemical staining and found expression of H3Cit (Figure 2B) and MPO **(**Figure 2C **)** in ccRCC.

**TABLE 1:** Clinicopathological features of 183 patients with metastatic ccRCC.

	All patients (%) (*n* = 183)	Targeted therapy group (%) (*n* = 150)	IO‐TKI group (%) (*n* = 33)
Age			
<60 years	110 (60.1)	89 (59.3)	21 (63.6)
≧60 years	73 (39.9)	61 (40.7)	12 (36.4)
Sex			
Male	154 (84.2)	123 (82.0)	31 (93.9)
T stage (initial diagnosis)			
T1	63 (34.4)	48 (32.0)	15 (45.4)
T2	18 (9.8)	12 (8.0)	6 (18.2)
T3	77 (42.1)	67 (44.7)	10 (30.3)
T4	25 (13.7)	23 (15.3)	2 (6.1)
N stage (initial diagnosis)			
N0	152 (83.1)	122 (81.3)	30 (90.9)
N1	31 (16.9)	28 (81.3)	3 (9.1)
M stage (initial diagnosis)			
M0	93 (50.9)	73 (48.7)	20 (60.6)
M1	90 (49.1)	77 (51.3)	13 (39.4)
ISUP grade			
G1/G2	44 (24.0)	38 (25.3)	6 (18.2)
G3/G4	139 (76.0)	112 (74.7)	27 (81.8)
Median tumor size (cm)	6.0	6.5	5.5
Venous tumor thrombus			
Yes	38 (20.8)	33 (22.0)	5 (15.2)
No	145 (79.2)	117 (78.0)	28 (84.8)
Time from diagnosis to treatment			
<1 year	136 (74.3)	104 (69.3)	32 (97.0)
≥1 year	47 (25.7)	46 (30.7)	1 (3.0)
KPS			
<80	27 (14.8)	24 (16.0)	3 (90.9)
≥80	156 (85.2)	126 (84.0)	30 (9.1)
IMDC			
Favorable‐risk	40 (21.9)	39 (26.0)	1 (3.0)
Intermediate‐risk	88 (48.1)	61 (40.7)	27 (81.8)
High‐risk	55 (30.0)	50 (33.3)	5 (15.2)
Involved metastatic sites			
Lung	132 (72.1)	114 (76.0)	18 (54.5)
Bone	46 (25.1)	39 (26.0)	7 (21.2)
Liver	8 (4.4)	8 (5.3)	0
Brain	4 (2.2)	3 (2.0)	1 (3.0)
Adrenal gland	11 (6.0)	6 (4.0)	5 (15.2)
Pleura	2 (1.1)	1 (0.7)	1 (3.0)
Pancreas	9 (4.9)	9 (6.0)	0
Median pretreatment NLR	2.6	2.6	2.6
Median OS (mo)	29.6	32.6	Not reached
Median PFS (mo)	12.2	14.0	9.0
Drug regimens			
Sunitinib	117 (63.9)	117 (78.0)	
Pazopanib	18 (9.8)	18 (12.0)	
Axitinib	15 (8.2)	15 (10.0)	
Pembrolizumab+axitinib	29 (15.8)		29 (87.8)
Tislelizumab+axitinib	4 (2.2)		4 (12.1)
Treatment sequence			
First‐line	150 (82.0)	135 (73.8)	15 (8.2)
Second‐line	33 (18.0)	15 (8.2)	18 (9.8)
Objective response rate			
Complete response	8 (4.4)	8 (5.3)	0
Partial response	71 (38.8)	59 (39.3)	12 (36.4)
Stable disease	79 (43.2)	63 (42.0)	16 (48.5)
Progression disease	25 (13.7)	20 (13.3)	5 (15.2)

**FIGURE 3:**
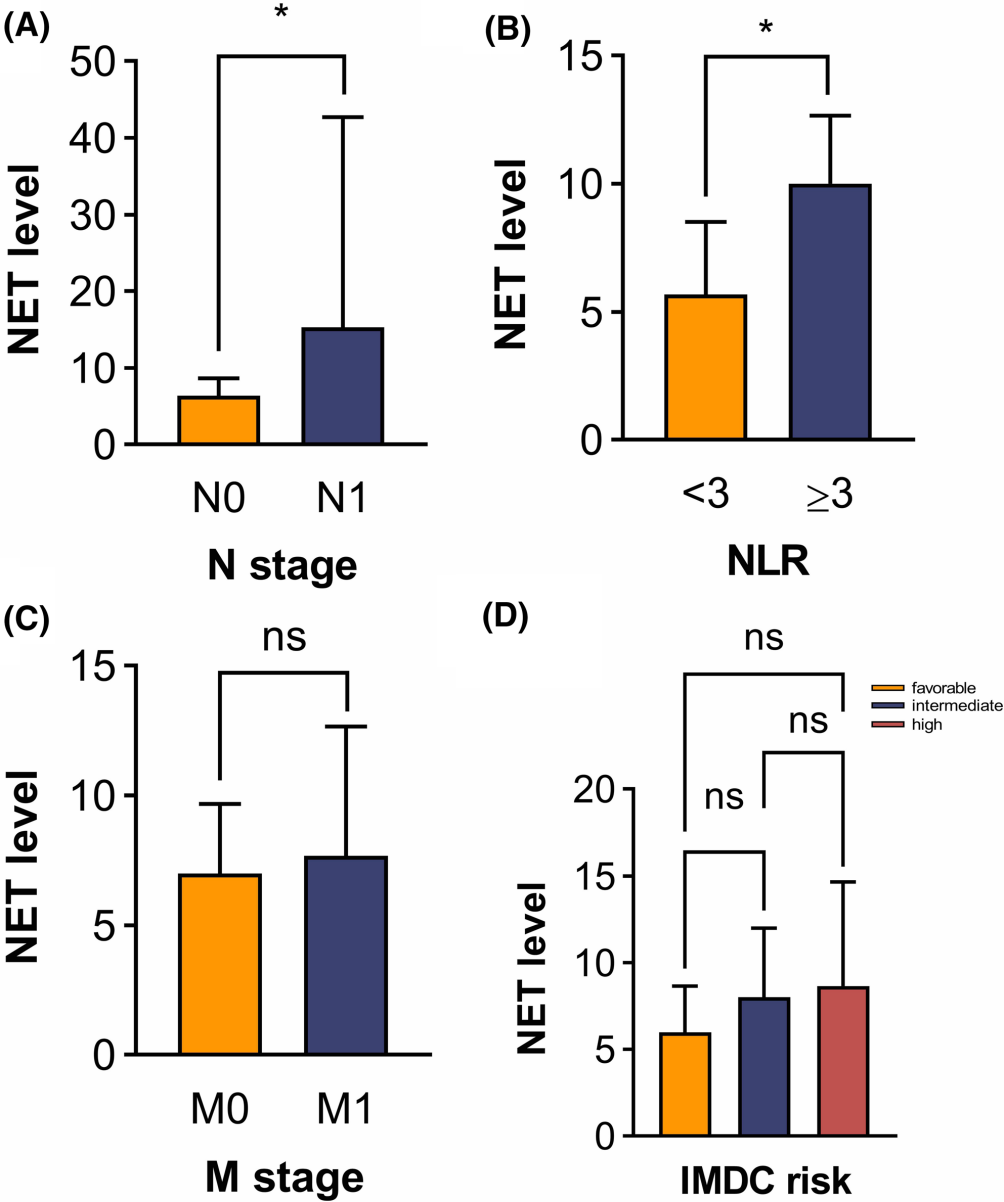
Association of NET with clinicopathological features in ccRCC. (A) N stage (B) Neutrophil‐to‐lymphocyte ratio (NLR) (C) M stage (D) IMDC risk I. (*p < 0.05, ***p < 0.001, ns: not significant).

By immunofluorescence staining, we analyzed NET levels in metastatic ccRCC. Notably, MPO expression was mainly located on the cell membrane, whereas H3Cit was located in the nucleus (Figure 2D). Figure 2E shows H3Cit and MPO levels in matched tumor tissue and para‐tumor tissue of the same patient. Among all patients, NET was more activated in tumor tissue than in adjacent normal tissue (*p* < 0.001). By calculating NET(+) cell counts, we analyzed the correlation between NET level in tumor tissue and clinicopathological features (Figure 3). The results suggested that a higher level of NET was significantly correlated with tumor N stage (*p* = 0.02), and NLR (*p* = 0.02), but there was no significant correlation with age, ISUP grade, tumor T stage, tumor M stage, venous tumor thrombus, and IMDC risk (*p* > 0.05). The full results of clinicopathological features are presented in Figure S2.

### 3.2 NET predicts systemic therapy efficacy in patients with metastatic ccRCC

**FIGURE 4:**
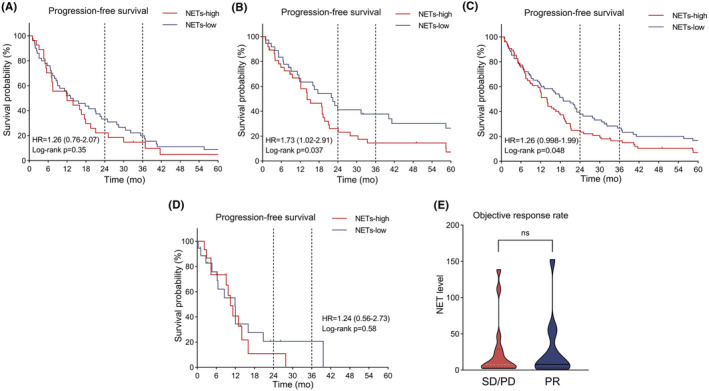
Association between NET and efficacy of targeted therapy/IO‐TKI in metastatic ccRCC. (A) Progression‐free survival (PFS) in the TKI group with synchronous metastasis (B) PFS in the TKI group with metachronous metastasis (C) PFS in all patients of the TKI group (D) PFS in the IO‐TKI group (E) Objective response rate (ORR) in the IO‐TKI group (ns: not significant).

The median PFS was 14.0 months and the median OS was 32.6 months in the TKI group. The median PFS was 9.0 months, and the median OS was not reached in the IO‐TKI group. For the targeted therapy group with synchronous metastasis **(**Figure 4A **)**, no correlation between the NET level and PFS was observed (HR 1.26 [95% CI 0.76–2.07], log‐rank *p* = 0.35). Notably, in the TKI group with metachronous metastasis **(**Figure 4B **)**, the lower NET level was associated with better PFS (HR 1.73 [95% CI 1.02–2.91], log‐rank *p* = 0.037). Overall, low NET level indicated better PFS in all patients with targeted therapy (HR 1.23 [95% CI 0.998–1.99], log‐rank *p* = 0.048) **(**Figure 4C **)**. However, no correlation between NET level and PFS was observed in the IO‐TKI group (HR 1.24 [95% CI 0.56–2.73], log‐rank *p* = 0.58) **(**Figures 4D **)**. Further analysis of ORR found no significant correlation with NET levels (*p* = 0.95) (Figure 4E). COX risk regression analysis was performed in the targeted therapy group with metachronous metastasis, and potential risk factors were included. As shown in Table 2, NET(+) cell counts in tumor tissue could be a predictor of the efficacy (HR 1.742 [95% CI 1.026–2.960], *p* = 0.040), while age, IMDC risk, NLR, T stage, N stage, ISUP grade, and venous tumor thrombus were not significant risk factors (*p* > 0.05).

**TABLE 2:** COX analysis of targeted therapy group with metachronous metastasis (*p<0.05).

Variation	Univariate analysis	
	HR (95% CI)	*p* value
Age	0.995 (0.970–1.021)	0.714
IMDC risk (high‐risk vs intermediate‐risk vs. favorable‐risk)	1.165 (0.791–1.716)	0.440
NLR	1.009 (0.961–1.059)	0.720
NET(+) cell counts (>7.3 vs. ≤7.3)	1.742 (1.026–2.960)	0.040*
T stage (T3/T4 vs. T1/T2)	1.050 (0.624–1.767)	0.855
N stage (N1 vs. N0)	2.645 (0.937–7.469)	0.066
ISUP grade (G3/G4 vs. G1/G2)	0.764 (0.442–1.321)	0.336
Tumor thrombus (yes vs. no)	0.778 (0.392–1.545)	0.473

### 3.3 NET predicts prognosis in patients with metastatic ccRCC

**FIGURE 5:**
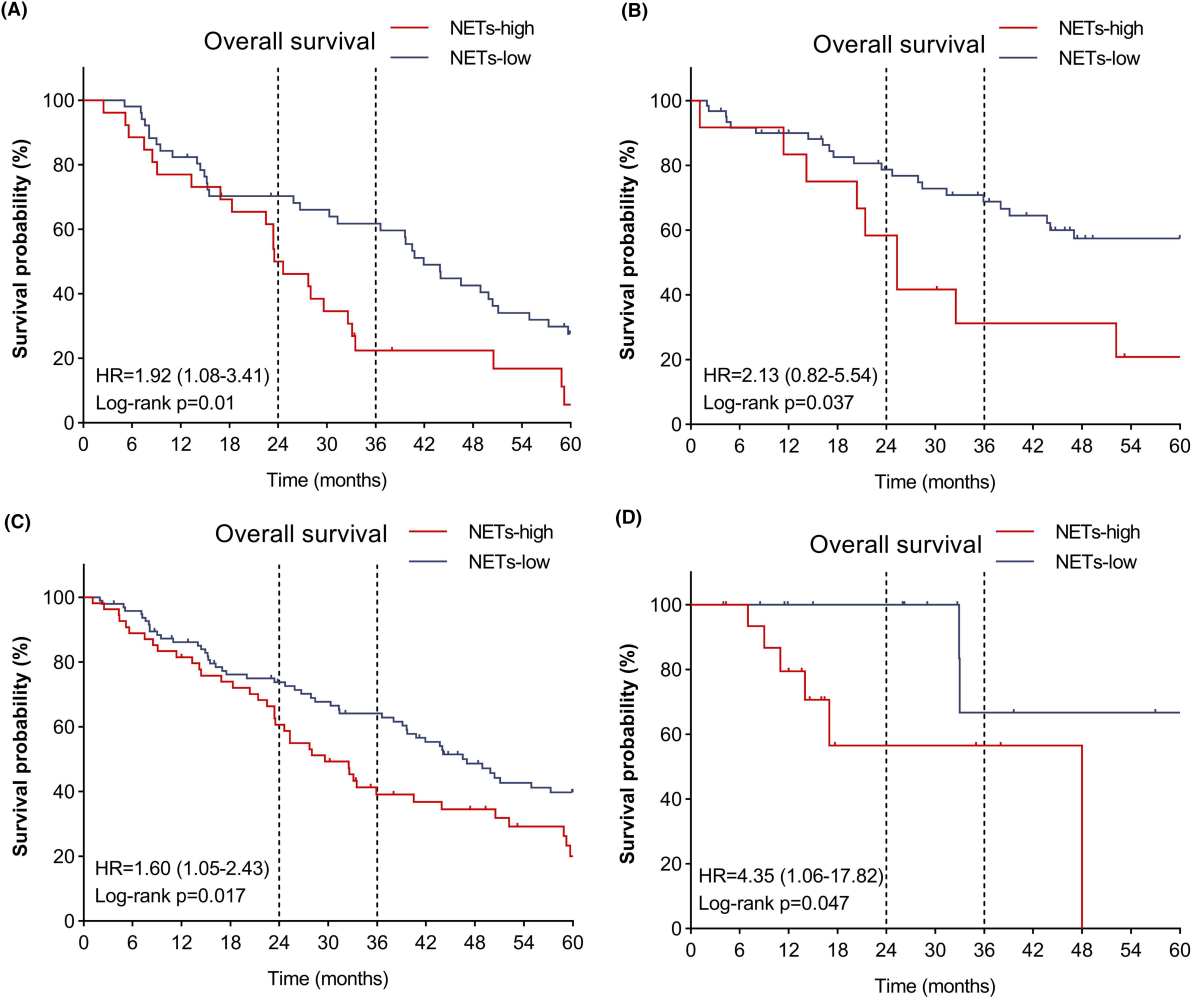
Association between NET and survival prognosis in ccRCC. (A) Overall survival (OS) in the TKI group with synchronous metastasis (B) OS in the TKI group with metachronous metastasis (C) OS in the TKI group (D) OS in the IO‐TKI group.

We further analyzed the OS in patients with metastatic ccRCC, with a median OS of 29.6 months for all patients, 27.7 months for patients with synchronous metastasis, and 31.4 months for patients with metachronous metastasis. As shown in Figure 5A, high NET levels in tumor tissue were associated with poor OS prognosis in the TKI group with synchronous metastasis (HR 1.92 [95% CI 1.08–3.41], log‐rank *p* = 0.01). Consistently, this association was observed in the TKI group with metachronous metastasis (HR 2.13 [95% CI 0.82–5.54], log‐rank *p* = 0.037) (Figure 5B). By analyzing the OS in separated groups of different treatments, we found that activated NET level was associated with poor OS in both TKI (HR 1.60 [95% CI 1.05–2.43], log‐rank *p* = 0.017) (Figure 5C) and IO‐TKI groups (HR 4.35 [95% CI 1.06–17.82], log‐rank *p* = 0.047) **(**Figure 5D **)**. A sensitivity analysis with patients who received first‐line therapy is presented in Figure S3. For the first‐line TKI group (*n* = 135), we found no significant difference in PFS for NETs‐high and NETs‐low groups (HR 1.92 [95% CI 1.08–3.41], log‐rank *p* = 0.10), while the OS curves were different (HR 1.62 [95% CI 1.04–2.52], log‐rank *p* = 0.02). When analyzing the first‐line IO‐TKI group (*n* = 15), no difference was found in PFS (HR 1.22 [95% CI 0.37–4.08], log‐rank *p* = 0.75) and OS (HR 0.81 [95% CI 0.05–13.27], log‐rank *p* = 0.88) due to the small sample size in the subgroup.

The univariate COX regression analysis suggested that tumor N stage (*p* = 0.017), M stage (*p* = 0.039), IMDC risk (*p* = 0.003), and NET level (*p* = 0.004) were the risk factors for OS (Table 3). By including statistically significant parameters from univariate analysis into multivariate analysis, only IMDC risk (HR 1.462 [95% CI 1.030–2.075], *p* = 0.033) and NET level (HR 1.733 [95% CI 1.165–2.579], *p* = 0.007) were independent prognostic risk factors for OS in patients with metastatic ccRCC.

**TABLE 3:** COX analysis of overall survival in patients with metastatic ccRCC (*p<0.05).

Variation	Univariate analysis		Multivariate analysis	
	HR (95% CI)	*p* value	HR (95% CI)	*p* value
Age	1.009 (0.990–1.028)	0.367		
T stage (T3/T4 vs. T1/T2)	1.120 (0.947–1.326)	0.185		
N stage (N1 vs. N0)	1.744 (1.104–2.755)	0.017*	1.155 (0.694–1.922)	0.578
M stage (M0 vs. M1)	1.496 (1.021–2.191)	0.039*	1.038 (0.650–1.656)	0.876
ISUP grade (G3/G4 vs. G1/G2)	1.080 (0.685–1.703)	0.739		
Tumor thrombus (yes vs. no)	0.950 (0.588–1.535)	0.834		
Treatment (TKI vs. IO‐TKI)	0.687 (0.331–1.424)	0.313		
NLR	1.011 (0.982–1.040)	0.476		
IMDC risk (high‐risk vs. intermediate‐risk vs. favorable‐risk)	1.506 (1.153–1.969)	0.003*	1.462 (1.030‐2.075)	0.033*
NET(+) cell counts(≤12.0 vs. >12.0)	1.759 (1.201‐2.575)	0.004*	1.733 (1.165‐2.579)	0.007*

## 4 DISCUSSION

Consistent with neutrophil properties, NET can induce cancer‐promoting or cancer‐suppressing effects in tumors. This study mainly explored the cancer‐promoting effect of NET in metastatic ccRCC and revealed the relationship between NET and clinicopathological features. By analyzing the biomarker level of NET in tumor and para‐tumor tissue, we found NET could be a predictor of drug efficacy and prognosis.

H3Cit and MPO are specific markers of NET and are used to quantify NET in tissue and peripheral blood. Previous studies in pan‐cancer indicated that H3Cit and MPO were effective prognostic markers.^20^, ^27^ By detecting MPO‐DNA, NE‐DNA, and H3Cit with enzyme‐linked immunosorbent assay (ELISA) in serum and plasma, NET was correlated with poor tumor staging, poor prognosis, and high risk of metastasis.^28^, ^29^, ^30^ Compared with using H3Cit or MPO as biomarker alone, we quantified NET with cell counts co‐expressed with H3Cit and MPO simultaneously, with better reliability. Immunofluorescence staining showed that NET in tumor tissue was more active than adjacent normal tissue. Neutrophil infiltration in kidney cancer has been considered a signature of poor prognosis.^31^ As one of the mechanisms by which neutrophils function, NET activity is consistent with neutrophil infiltration. Notably, tissue NET level was associated with tumor N stage at initial diagnosis. Secondary lymph node organs serve as sites for the colonization and proliferation of immune cells, providing a microenvironment for the interaction of innate and adaptive immune systems. In the peripheral circulation, neutrophils can be recruited in tumor‐draining lymph nodes and NET can be induced when stimulated by chemokines such as IL‐17.^32^, ^33^ To date, the underlying mechanism of lymph node metastasis in kidney cancer remains unclear, and NET might be a potential trigger. In addition, there was no significant correlation between NET and initial M stage (synchronous metastasis vs metachronous metastasis), indicating that the tissue NET activity of primary tumor might not be a key inducer in metastasis. However, NET in peripheral blood promoted hematogenous metastasis by enveloping CTCs and assisting them in survival and migration.^19^

The recommended first‐line treatment for metastatic ccRCC is IO‐TKI, but there are no effective predictors of drug efficacy. Labriola et al analyzed tumor specimens of metastatic ccRCC treated with IO and found no correlation between therapy response and traditional efficacy markers such as TMB and PD‐L1 expression.^12^ As one of the parameters of IMDC risk, neutrophil counts are of great significance for patient prognosis stratification and guiding treatment. We assumed that NET might be a potential predictor of drug efficacy. Chen et al. constructed a risk model of six NET‐related genes in head and neck squamous cell carcinoma, and the low‐risk group received more benefits from anti‐PD‐1 therapy than the high‐risk patients.^34^ Interestingly, tissue NET level was also a negative predictor of OS with both TKI and IO‐TKI for metastatic ccRCC. However, no association between PFS and ORR was observed with NET in the IO‐TKI group. In patients who received IO+TKI therapy, no difference in PFS was observed between the NETs‐high and NETs‐low groups, while the OS in the NETs‐low group was significantly longer compared to the NETs‐high group. The proportion of patients who received first‐line and second‐line treatment in the IO‐TKI group was close, which might affect the difference in PFS. Another possible explanation is that NET has an effect on prognosis in RCC beyond the medical treatment.^20^, ^27^ Further exploration with long‐time follow‐up in large‐scale cohorts is needed. A total of 33 patients were enrolled in the IO‐TKI group, with a small sample size and ratio of first‐line versus second‐line at 5:6, and the results of this study may be altered by enlarging the sample size and optimizing the sequence of treatment. Santoni et al. revealed that host immune status was associated with VEGF‐TKI efficacy in metastatic kidney cancer that occurred more than 5 years after radical nephrectomy, with a median PFS of 25.1 months (95% CI 11.7—NA) for NLR <3 patients and 15.8 months (95% CI 7.1–31.3) for NLR ≥ 3.^35^ Consistently, elevated NET level with metachronous metastasis was associated with poor PFS in the TKI group. Therefore, NET levels in primary tumor tissue may reflect the host immune response to tumor, while patients with active local immune status respond better to TKIs. In the clinical dissection of tumor, tumor tissue NET can be assessed to guide the potential dose of targeted agents. The IMDC risk score stratifies patients according to median OS and guided treatment. Active NET in tumor tissue was associated with poor OS in metastatic ccRCC. Multivariate COX analysis showed that NET and IMDC had a greater impact on prognosis than risk factors such as primary tumor stage, histological grade, and venous tumor thrombus. After progression to distant metastasis, NET reflecting the host immune response is an effective predictor.

In this study, NET is more predictable for PFS and OS in the TKI group than in the IO‐TKI group. Immune checkpoint blockade of PD‐1 and PD‐L1 can restore the function and survival of exhausted CD8+ T cells, and then activate the cytotoxicity toward tumor cells.^36^ NET can interact with infiltrating CD8+ T cells to promote an immunosuppressive tumor microenvironment. Neutrophils isolated from humans could form NET and induce exhaustion and dysfunction of CD8+ T cells.^37^ For colorectal cancer, DNase I inhibits NET formation in vivo and reverses anti‐PD‐1 blockade resistance through increasing CD8+ T cell infiltration and cytotoxicity.^38^ To date, it remains unclear if NET is associated with drug resistance of VEGF‐TKI, though NET plays a role in promoting angiogenisis by interacting with angiogenic factors (VEGF‐A and angiopoietins).^39^, ^40^ Therefore, NET effects on immune checkpoint blockade with anti‐angiogenisis might be more complex and less predictable than using ICI or TKI alone.

Neutrophil infiltration is an indication of poor prognosis for ccRCC, and this study revealed NET as a potential role of neutrophils affecting the tumor microenvironment. With long‐time follow‐up of patient cohorts, NET can be a biomarker of systemic therapy efficacy. Some limitations of the research should be stated. First, the sample size of IO‐TKI cohort was small, and the ratio of first‐line and second‐line was 5:6, which might not represent the real‐world experience. Second, due to tissue necrosis and low staining quality after treatment, matching sample analysis before and after treatment was missing. Third, *p*‐value of 0.048 in the PFS analysis of all targeted therapy patients may alter conclusions when adjusting the sample size.

## 5 CONCLUSIONS

For metastatic ccRCC, NET was more active in tumor tissue than in para‐tumor tissue. The lower level of tumor tissue NET predicts better drug efficacy in patients treated with TKI and IO‐TKI. NET could be the mechanism of neutrophil‐promoting tumor, and NET formation in ccRCC tumor tissue was associated with poor clinical outcomes. Quantifying NET may optimize the treatment strategy for kidney cancer.

## AUTHOR CONTRIBUTIONS

**Ruiyang Xie:** Conceptualization (lead); formal analysis (lead); methodology (equal); software (equal); validation (equal); writing – original draft (equal); writing – review and editing (equal). **Bingqing Shang:** Investigation (equal); methodology (equal); software (equal); writing – original draft (equal). **Hongzhe Shi:** Methodology (supporting); software (supporting); supervision (equal); writing – review and editing (supporting). **Xingang Bi:** Software (supporting); supervision (equal); writing – review and editing (supporting). **Yan Song:** Conceptualization (supporting); data curation (supporting); supervision (equal). **Wang Qu:** Conceptualization (supporting); data curation (supporting); supervision (supporting). **Hongsong Bai:** Data curation (supporting); methodology (supporting); writing – review and editing (supporting). **Linjun Hu:** Conceptualization (supporting); methodology (supporting); writing – review and editing (supporting). **Jie Wu:** Data curation (supporting); formal analysis (supporting); software (equal); writing – review and editing (supporting). **Honglei Cui:** Conceptualization (supporting); data curation (supporting); methodology (supporting). **Gan Du:** Conceptualization (supporting); data curation (supporting); methodology (supporting); writing – review and editing (supporting). **Lei Guo:** Conceptualization (equal); data curation (equal); software (equal); supervision (equal); writing – review and editing (equal). **Shan Zheng:** Data curation (equal); methodology (equal); supervision (equal); writing – review and editing (equal). **Jianming Ying:** Conceptualization (equal); data curation (equal); methodology (equal); supervision (supporting); writing – review and editing (supporting). **Changling Li:** Data curation (supporting); methodology (supporting); supervision (supporting); writing – review and editing (supporting). **Jianhui Ma:** Conceptualization (equal); methodology (supporting); supervision (supporting); writing – review and editing (supporting). **Aiping Zhou:** Conceptualization (equal); methodology (equal); supervision (supporting); writing – review and editing (supporting). **Jianzhong Shou:** Conceptualization (equal); funding acquisition (lead); methodology (equal); project administration (equal); resources (equal); supervision (lead); writing – review and editing (equal).

## FUNDING INFORMATION

This study is funded by the National Natural Science Foundation of China (ID: 82072837), Beijing Municipal Natural Science Foundation (ID: 7212083), and Beijing Hope Run Special Fund of Cancer Foundation of China (ID: LC2018L02).

## CONFLICT OF INTEREST STATEMENT

No potential conflict of interest was reported by the authors.

## ETHICS STATEMENT

Approval of the research protocol by an Institutional Reviewer Board: This retrospective study was approved by the National Cancer Center Ethics Committee (Ethics Approval Number: NCC2020C‐122).

Informed Consent: This retrospective study waived informed consent.

Registry and the Registration No. of the study/trial: N/A.

Animal Studies: N/A.

## Supporting information


Figure S1.
Figure S2.
Figure S3.

## Data Availability

The authors confirm that the data supporting the findings of this study are available within the article and its supplementary materials.
