# Supplementary material for: Neutrophil extracellular traps in relationship to efficacy of systemic therapy for metastatic renal cell carcinoma
Source: Cancer Med. 2023 Nov 28;12(24):21807–19. doi: 10.1002/cam4.6748 (PMC10757093; doi:10.1002/cam4.6748)

**Neutrophil extracellular traps in relationship to efficacy of systemic therapy for metastatic renal cell carcinoma**

Supplementary materials

**Supplementary Figure S1.** Nine wax blocks of TMA


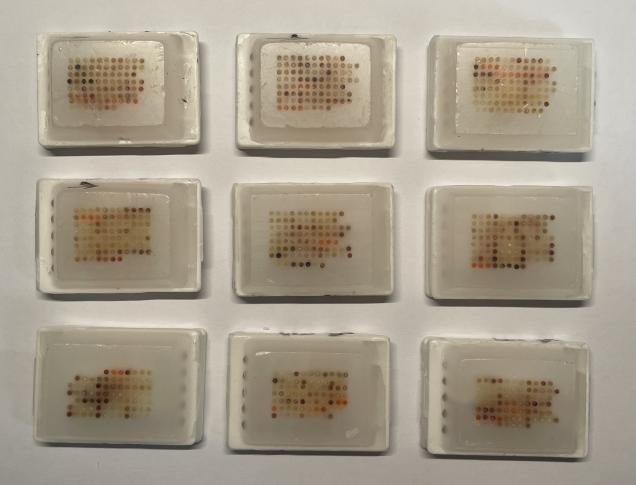


**Supplementary Figure S2.** Association of NET with clinicopathological features in ccRCC


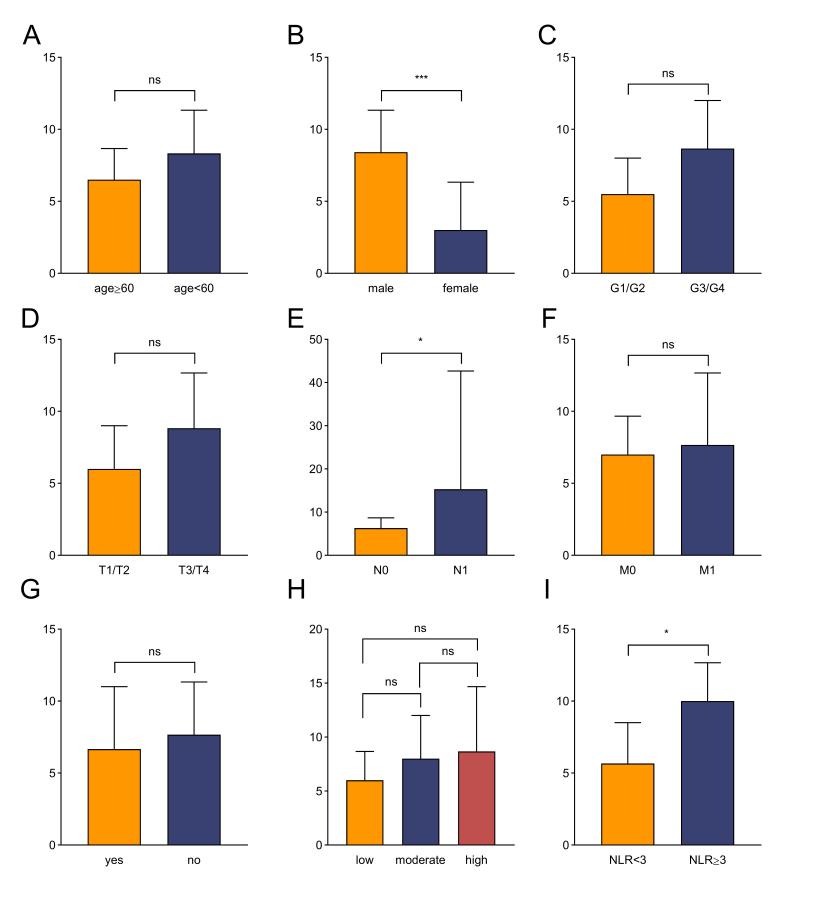


**Supplementary Figure S3.** Sensitivity analysis of NET in patients who received first-line TKI and IO-TKI treatment. **A.**Progression-free survival (PFS) in TKI group **B.** Overall survival (OS) in TKI group **C.** PFS in all patients of IO-TKI group **D.** OS in IO-TKI group


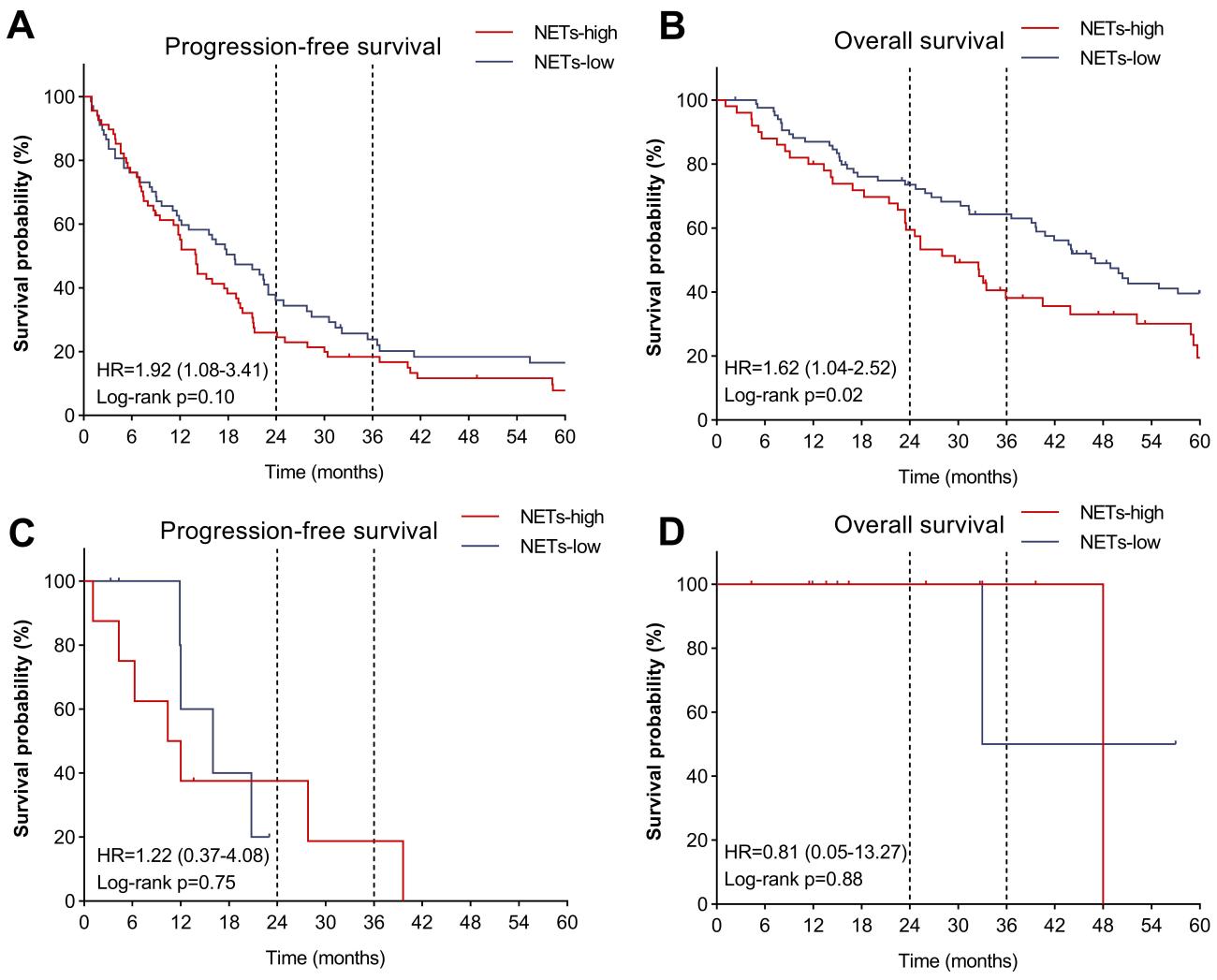

Supplement: Supplementary file 1 — Figure S1. Figure S2. Figure S3. [file CAM4-12-21807-s001.docx]
